# Bioaugmentation with native *Bacillus* strains enhance nitrate and nitrite removal and reshape microbiomes in low-salinity shrimp cultures: Elucidating genetic mechanisms

**DOI:** 10.1371/journal.pone.0339620

**Published:** 2026-01-09

**Authors:** Dámaris Adelaida Esquén Bayona, Delia Talledo Ancajima, Dorian Adriano Cadena, Luz Dominguez-Mendoza, Sebastian Leyva, Mia Mariana Somocurcio Zambrano, David Edilberto Saldarriaga Yacila, Pabulo Henrique Rampelotto, Frank Lino Guzman Escudero, Benoit Mathieu Diringer

**Affiliations:** 1 Laboratory of Biomolecules, Faculty of Health Sciences, Universidad Peruana de Ciencias Aplicadas (UPC), Lima, Perú; 2 Incabiotec SAC, Tumbes, Perú; 3 Universidad Nacional de Tumbes, Tumbes, Perú; 4 Bioinformatics and Biostatistics Core Facility, Federal University of Rio Grande do Sul, Porto Alegre, Brazil; 5 Concepto Azul South Australia, Estero Salado, Guayaquil, Ecuador; Universidade Catolica Portuguesa, PORTUGAL

## Abstract

The intensification of shrimp farming contributes to the accumulation of toxic nitrogen compounds, which in turn affect productivity and complicates water quality management, especially under conditions of reduced salinity. This study evaluated the effects of 25% (CO, T2) and 50% (T1) water exchange, in combination with a bioaugmentation treatment using native *Bacillus* (T2), on nitrogen compound concentrations and bacterial community structure in *Litopenaeus vannamei* culture under reduced salinity conditions (4ppt). The results demonstrated that treatment with native *Bacillus megaterium* and *Bacillus paralicheniformis* (T2) leads to a significant reduction in nitrite and nitrate concentrations, reaching nearly 0 mg/L from day 4. In contrast, T1 and CO treatments showed markedly higher concentration, reaching up to 5 mg/mL and 160 mg/L, respectively. The full-length 16S rRNA gene used for the metataxonomic analysis revealed changes in bacterial composition towards species with nitrifying and probiotic potential, with native *Bacillus* strains detected exclusively in T2. In addition, a reduction in bacterial diversity was detected, and significant differences were observed between the bacterial communities of T2 and those of T1 and CO (p = 0.001, *R*^*2*^* = *0,328). The shotgun analysis further revealed a higher abundance of enzymes related to nitrification and dissimilatory nitrate reduction to ammonium in T2 treatment. The results highlighted the active involvement of Gram positive – *Bacillus* and Gram-negative bacteria such as *Shewanella* and *Psychrobacter*, and suggesting heterotrophic nitrification and aerobic denitrification. Overall, native *B. megaterium* and *B. paralicheniformis* provided an effective bioaugmentation strategy for the managing nitrate and nitrite in low-salinity shrimp farming, providing an eco-friendly alternative that may enhance productivity and reduce the industry´s water footprint.

## 1. Introduction

The “white shrimp” *Litopenaeus vannamei* Boone, L. 1931 is the most important aquaculture species produced globally with a total production of 6.8 million tons in 2022 [1]. In recent decades, the intensification of *L. vannamei* farming has led to the development of advanced technologies to address both water scarcity and disease management challenges [2]. Among these technologies, low salinity farming has also been proposed as an alternative to increase production and mitigate problems associated with the availability of coastal land and marine water diseases [3]. Studies have indicated optimal growth of *L. vannamei* in salinity levels as low as 5 ppt, allowing farming beyond traditional coastal [4,5]. However, shrimp tolerance to nitrogenous compounds such as ammonium, nitrite, and nitrate decreases under the previously mentioned conditions, posing a challenge for farm management, as these compounds negatively affect shrimp gills, leading to reduced food intake and slower growth [6–8]. To mitigate such risks, threshold concentrations have been established for low salinity farming: < 0.1 mg/L for ammonium, < 0.23 mg/L for nitrite, and between 0.2 and 10 mg/L for nitrate [9]. Water exchange is commonly implemented to control nitrogenous compounds, however it is costly and requires substantial operational effort. A daily water exchange of 10–15% is generally recommended to reduce organic loads and toxic metabolites [10]; while studies suggest that even higher rates, such as 25%, may be more effective at controlling nitrate accumulation [11].

An alternative approach involves autotrophic nitrifying and heterotrophic denitrifying microorganisms, which regulate the transformation and removal of ammonium, nitrite, and nitrate through ammonification, nitrification, and denitrification processes. Notably, some bacteria are capable of performing simultaneous heterotrophic nitrification and aerobic denitrification, offering a more efficient alternative for nitrogen removal [12,13]. Among these, *Bacillus* species, widely used as aquaculture probiotics, have received increasing attention with several strains demonstrating high nitrogen removal capacities in both saline and freshwater environment [14]. These bacteria improve water quality in farming systems through multiple pathways, including ammonification, nitrification, denitrification, and nitrogen fixation [15–18]. However, previous studies have also reported that bioaugmentation strategies in aquaculture systems can modify native microbial community structure and, under certain conditions, such shifts may create opportunities for the proliferation of opportunistic pathogens [19,20].

In this context, the use of microorganisms in shrimp farming is conceived as a controlled microbial intervention intended to modulate key biochemical processes and support the functional stability of the system. In addition to these considerations, most studies have focused on commercial strains tested under controlled conditions and some findings have reported inconsistent effects on water quality parameters [14,21], leaving a knowledge gap regarding how native *Bacillus* strains with heterotrophic nitrification and aerobic denitrification capacity affect microbial community dynamics, their ecological role within the aquaculture water microbiome, and which metabolic pathways are implicated under aquaculture conditions.

Moreover, recent studies suggest that nutrient regulation can significantly influence microbial communities in both terrestrial and aquatic environments. Recent work by Pan et al. [22] demonstrated that varying phosphorus levels modulate microbial communities and growth performance in livestock. Likewise, Tang et al. [23] found that salinity and nutrient levels, particularly nitrogen compounds, affect bacterial diversity and community composition in aquatic ecosystems. These studies suggest that nutrient management, including nitrogen compounds, may play a significant role in altering microbial dynamics in aquaculture systems. Nanopore sequencing technology has established itself as an effective tool for metataxonomic and metagenomic studies, allowing the exploration of cultivable and non-cultivable microbial communities [24–26]. Its long-read format is particularly advantageous for analyzing key processes such as nitrification, denitrification, and the synthesis of bacterial virulence factors in shrimp aquaculture systems [27,28].

In this study, we evaluate a bioaugmentation approach based on the application of native *Bacillus* strains in low-salinity shrimp culture. We hypothesize that native strains such as *B. megaterium* and *B. paralicheniformis*, previously shown to be effective in nitrogen compounds bioremediation under high-salinity conditions (30 ppt) [15], may also reduce levels of ammonium, nitrite, and nitrate under low-salinity conditions. To test this hypothesis, we assessed their efficiency in shrimp culture at 4ppt and evaluated their effect on the bacterial community through full-length 16S rRNA gene sequencing using Nanopore technology. Additionally, we performed shotgun metagenomic sequencing to identify the metabolic pathways involved in the degradation of nitrogen compounds.

## 2. Materials and methods

### 2.1. Acclimatization *L. vannamei to cultivation conditions*

Shrimp culture were carried out at the Aquatic Biotechnology Experimental Center of Puerto Pizarro (CEBAP) located in Tumbes, Peru (3°30’07“S 80°23’42”W). No specific permits were required for the use of farmed shrimp, and all work complied with institutional, national, and international research guidelines. The operation of CEBAP is officially authorized by the Peruvian Ministry of Production (PRODUCE). Microbiology and Molecular Biology experiments carried out by INCA BIOTEC S.A.C. are accredited by the National Council for Science, Technology, and Technological Innovation (CONCYTEC) of Peru (Resolutions Nº 082–2021-CONCYTEC-SDCTT and Nº 083–2021-CONCYTEC-SDCTT).

The shrimp were obtained from suppliers in Peru at the PL12 stage and were gradually acclimated using freshwater until a salinity of 4 ppt was reached in the culture. Prior to the trial, all shrimp were maintained under standard environmental conditions in the acclimatization area, which included continuous aeration, a temperature range of 25–30°C, a pH of approximately 8, and dissolved oxygen (DO) around 6 mg/L. Feeding was provided twice daily with *Nicovita Origin Organic* pellet 0.8 (≥ 45% protein) balanced feed. The culture water was pre-treated and disinfected with calcium hypochlorite (DAIG114320, PLUSCHLOR, China) and neutralized with sodium metabisulfite (7681-57-4, BASF, Germany).

### 2.2. Native strains

The native strains *Bacillus megaterium (SM4) and Bacillus paralicheniformis* (SM5) used in this study were previously isolated in June 2018 from mangrove soil at Puerto Pizarro (Peru, Tumbes, 3°30’05“S 80°23’43”W y 3°30’03”S 80°23’44”W). These strains were selected based on their high removal efficiency of total ammonia nitrogen (TAN), nitrite, and nitrate, demonstrating strong potential for nitrogen compound bioremediation in aquaculture systems [15].

### 2.3. Experimental design

Three experimental conditions (CO, T1, and T2) were evaluated, each with three replicates per condition. In the in vivo study, 100 shrimp at the P.L21 stage were randomly selected and placed in experimental tanks containing 40 L of water at a salinity of 4 ppt. Details of the experimental setup are provided in Table 1. In treatment T2, 1 liter of a bacterial suspension containing a mixture of *Bacillus megaterium* and *Bacillus paralicheniformis* was added every three days at a concentration of 0.5 x 10^8 cells/mL (previously mass-cultured in Trypticase Soy Broth (M290-500G, HIMEDIA, INDIA) supplemented with 2% NaCl and 3% sugar) along with 3.6 g of sugar (ABA2606, DULFINA, PERU) as a carbon source. The C/N ratio of 0.175 was calculated according to Boyd [9]. Water renewal was performed on days after the initiation of the bioassay.

**Table 1:** Experimental design of bioassay.

Treatment	% water replacement	*Bacillus* supplementation	number of bins	number of shrimp/bins
CO	25	No	3	100
T1	50	No	3	100
T2	25	Yes	3	100

Environmental parameters of the treatments were recorded daily. Temperature, oxygen, and pH were measured using a HANNA brand thermometer, and salinity was measured with a 100SAL model refractometer. Concentration of total ammonia nitrogen (TAN), nitrite, and nitrate was determined using an API colorimetric kit. Subsequently, 500 mL of water from each replicate was collected in sterile bags on days 02, 04, 07, 10, 13, 14, and 16 of the bioassay. Biological material was stored at −20ºC and transported under refrigerated conditions to the Incabiotec S.A.C. laboratory for processing.

### 2.4. DNA extraction and Nanopore sequencing

A pool was obtained by combining the three replicates from each treatment. Then, 150 mL from each pool was filtered using Gamma Sterile 0.22 µm filters in a vacuum pump model 622.11.001 [29]. The filters were cut into pieces and incubated with proteinase K (D3001-2–20, Zymo Research, USA) for 30 minutes at 37°C. DNA was extracted using the ZymoBIOMICS DNA Miniprep Kit (D4300, Zymo Research, USA) [30]. DNA concentration and quality were assessed using fluorometry, NP80 spectrophotometry, and agarose gel electrophoresis [31]. To validate the presence of bacterial DNA, PCR amplification of the full 16S rRNA gene was performed using the primers F27 5’-AGAGTTTGATCMTGGCTCAG-3’ and R1492 5’-TACGGYTACCTTGTTACGACTT-3’, generating an ~ 1500 bp product. The final 25 µL reaction mixture contained 1X reaction buffer, 2 mM MgSO4, 200 nM dNTPs, 0.36 µM of primers F27 and R1492 [32], 1 U of High Purity Taq DNA polymerase, and 50 ng of genomic DNA. The PCR conditions included an initial denaturation at 95°C for 5 minutes, followed by 35 cycles of denaturation at 95°C for 30 seconds, annealing at 57°C for 40 seconds, extension at 72°C for 1 minute, and a final post-extension at 72°C for 6 minutes. The obtained amplicons were electrophoresed on a 1.5% agarose gel and visualized with 1X SYBR Safe (S1 Fig).

For the preparation of the metataxonomic libraries, samples from days 02, 04, 07, 10, 13, 14, and 16 of the experiment were included, each coded with the treatment name followed by the collection day. These libraries were prepared using the 16S Barcoding Kit 24 V14 (SQK-16S114.24, ONT, UK), which amplifies the V1 to V9 regions of the 16S rRNA gene from 20ng of input DNA using PCR with LongAmp Hot Start Taq DNA Polymerase 1X Master Mix (M0534S, NEB, USA) [33].

On the other hand, the DNA collected on day 10 in CO and treatment T2 was used to prepare the shotgun libraries using the Native Barcoding Kit 24 V14 (SQK-NBD114.24, ONT, UK). A total of 400 ng of input DNA was used for DNA repair and end preparation with the NEBNext FFPE DNA Repair Mix and End Repair/dA-Tailing kits. The barcoding ligation was then performed with Blunt/TA Ligase Master Mix, and the native adapters were ligated using the NEBNext Quick T4 DNA Ligase kit. Subsequently, both library types were purified using AMPure XP magnetic beads (A63880, Beckman Coulter, USA) and quantified using the Qubit fluorometer with the Qubit dsDNA HS Assay Kit (Q32851, Thermo Fisher Scientific, USA). A total of 100 fmol of the purified metataxonomic libraries were loaded onto the Flow Cell R9.4.1 (FLO-MIN106D, ONT, UK), while the shotgun libraries were loaded onto R10.4.1 flow cells (FLO-MIN114, ONT, UK) according to the manufacturer’s recommendations [34]. Sequencing was carried out on the MinION Mk1B device with data collection configured to be demultiplexed in FAST5 format using the minKNOW software.

### 2.5. Bioinformatic analysis

FASTQ files were generated from the FAST5 files using the Guppy software (v6.5.7) in the basecalling configurations HAC (metataxonomic) and SUP (metagenomic). The quality of the obtained sequences was visualized using NanoPlot (v1.41.6) [35]. Adapter sequences were identified and removed using Porechop (v0.2.4) while NanoFilt (v2.8.0) [36] was used to remove the reads with a Phred quality score average of < 10 for metataxonomic sequences and < 12 for shotgun sequences. For metataxonomic data, taxonomic assignment and relative abundance data for each sample were performed using the EMU software (v3.4.5) [37] with its default database as the reference. These results, along with the sample metadata, were imported and combined into an object using the phyloseq package (v1.46.0) in R (v4.3.2), and alpha and beta diversity indices were calculated [38]. For diversity metrics calculations, the sequences of each sample were rarified considering the sample with the least number of sequences. In the case of beta diversity, Bray-Curtis dissimilarity was used [39].

For shotgun data, FASTQ files for each treatment were analyzed using the SqueezeMeta pipeline (v1.6.3) [40,41] in sequential mode, and the obtained results were imported and analyzed in R (v4.3.2) using the SQMTools package (v1.6.3). The abundance data for each ORF, expressed as ORF per million (OPM), were filtered using Python (v3.12.3) to obtain genes involved in nitrite reduction and denitrification via KEGG orthology. Subsequently, data were exported to GraphPad Prism (v10.2.2) for the generation of a heatmap at the genus, family, or order level, grouping similar taxonomies together.

### 2.6. Statistical analysis

Concentration values of TAN (mg/L), nitrite (mg/L) and nitrate (mg/L) were recorded during each treatment and used in the statistical analysis. To determine possible significant differences between treatments, one-way ANOVA and the Tukey post hoc test were executed using the IBM SPSS statistics 21 program. For data sets not meeting the assumptions of normal distribution or homogeneity of variances, the nonparametric Kruskal-Wallis and Welch’s ANOVA tests were applied, respectively. These analyses were performed in R Studio (R.4.3.3).

In beta diversity analysis, permutational variance (PERMANOVA) was performed using the “adonis” function from the pairwiseAdonis package (v0.4) [39] to evaluate the impact of different treatments on microbial composition. A significance level of 0.05 was considered for all statistical tests applied (S9 Table).

### 2.7. Data deposition

The Nanopore sequencing reads are available in the NCBI Sequence Read Archive (SRA) under BioProject ID PRJNA1203271, with accession numbers SRR31851672-SRR31851692 for metataxonomic data, and SRR31842143-SRR31842144 for shotgun data.

## 3. Results

In this study, the effectiveness of water exchange treatment was evaluated in comparison to the application of bioremediating bacteria (*Bacillus megaterium* and *Bacillus paralicheniformis)* in reducing the concentration of toxic nitrogenous compounds in the culture water.

### 3.1. Increased water exchange effectiveness and the application of native *Bacillus* on nitrogenous compounds

**Fig 1:**
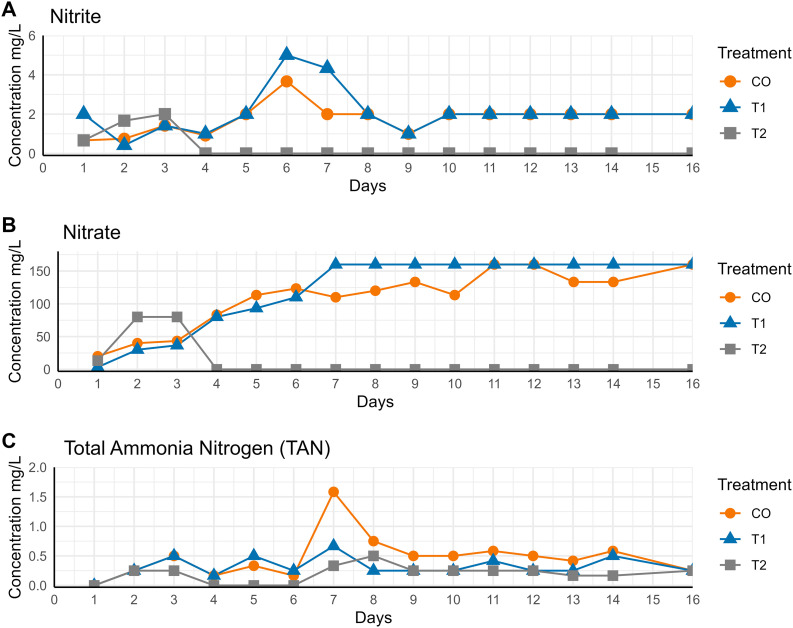
Concentration of nitrogen compounds in water replacement compared to native *Bacillus* treatment. The concentration (mg/L) of nitrite **(A)**, nitrate **(B)** and total ammonia nitrogen – TAN **(C)** were measured in low salinity culture water of shrimp *Litopennaeus vannamei* through 15 days. CO represents the control group with 25% water replacement, T1 represents the treatment with 50% water replacement, and T2 the treatment with 25% water replacement combined with native *Bacillus* strains (*Bacillus megaterium* and *Bacillus paralicheniformis*). Water replacements were carried out on days 3, 6, 10, and 13. Data are presented as means of n = 3 independent replicates.

The results indicated that treatment T2, which involved the application of native *B. megaterium* and *B. paralicheniformis* strains resulted in significantly lower nitrite concentrations (p < 0.05) from day 4 onward, in comparison to T1 and the control (CO), where concentrations ranged between 0.92 mg/L and 5 mg/L. Nitrite concentrations remained at 0 mg/L from day 4 onward (Fig 1A). In treatment T2, nitrate levels declined to 0 mg/L by day 6 and remained undetectable for the remainder of the experimental period, in contrast to treatments T1 and CO, where concentrations persisted between 110 and 160 mg/L (Fig 1B). Total ammonia nitrogen (TAN) concentrations, were significantly lower in treatment T2 on days 3, 5, and 6 (p < 0.05). From day 7 onward, TAN levels stabilized across all treatments, ranging from 0.17 to 0.50 mg/L, with no significant differences observed (p > 0.05) (Fig 1C, S1 table). Notably, no shrimp mortality was recorded in any of the treatments – including CO, T1, or T2, throughout the 16-day experimental period.

### 3.2. Full-length 16S rRNA gene-targeted analysis

To ensure both the quantity and integrity of the genetic material extracted from the shrimp farming water, a DNA extraction protocol using water filtration was employed. This method yielded DNA concentrations ranging from 100 ng/µL to 800 ng/µL after filtering 150 mL of water, with 260/280 ratios between 1.8 and 1.9 and 260/230 ratios between 1.8 and 2.4. Agarose gel electrophoresis revealed genomic DNA fragments larger than 10 kb, with no observable smears. PCR targeting the 16S rRNA gene produced bands of approximately 1500 bp, confirming that the in-house protocol generates high-quality DNA suitable for TGS sequencing (S1 and S2 Fig, S2 Table). After sequencing, a total of 773,013 reads were obtained across all samples, with an average quality score of 12.73. Following adapters and application of quality filters, 507,114 clean reads remained. The average number of clean reads per sample was 24,148, with the highest and lowest read counts being 59,495 and 10,614, respectively. Using these reads, EMU program identified 637 ASVs, taxonomically classified to the species level, with a minimum abundance of 10 reads in all samples (S3 Table).

### 3.3. Composition and distribution of bacterial communities

**Fig 2:**
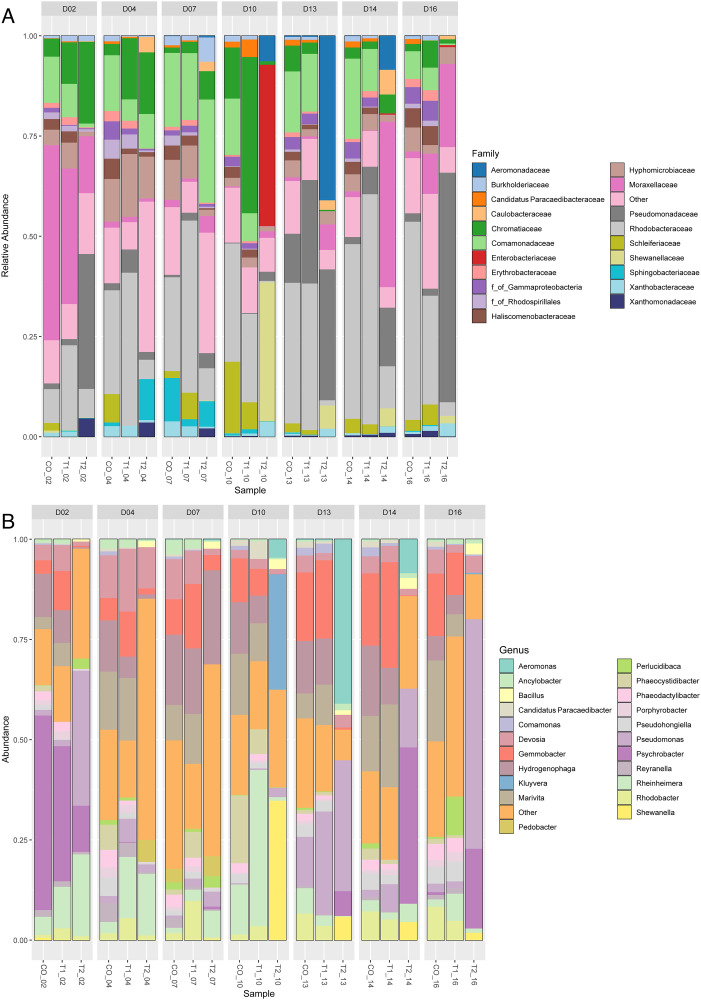
Relative abundances of the most representative bacteria detected by 16S rRNA gene amplicon sequencing. **A.** Data at the family level. **B.** Data at the genus level. Each bar represents specific conditions (CO, T1, and T2), and each box corresponds to a particular day.

The composition of bacterial communities in the culture ponds was included a total of 155 families distributed across the three treatments. The families with the highest relative abundance in the control (CO) were Rhodobacteraceae (8.46%−49.44%), Comamonadaceae (6.91%−19.99%), and Moraxellaceae (0.31%−48.65%). A notable abundance of Hyphomicrobiaceae was also observed (2.14%−10.65%). Similarly, in treatment T1, which involved the highest water exchange rate, the most abundant families were Rhodobacteraceae (21.12%−57.40%), Comamonadaceae (5.35%−16.69%), Moraxellaceae (0.15%−33.81%), and Chromatiaceae (1.84%−38.96%). In treatment T2, the predominant families were Pseudomonadaceae (1.94%−57.27%), Moraxellaceae (0.76%−41.30%), and Aeromonadaceae (0.07%−41.04%). The family Moraxellaceae was particularly noteworthy due to its wide distribution across CO and both treatments (Fig 2A, S3 Fig, S4 Table).

In CO a total of 202 genera were identified, with the most abundant being *Hydrogenophaga* (6.10%–17.56%), *Marivita* (3.02%–20.16%), *Gemmobacter* (3.36%–18.04%), *Psychrobacter* (0.68%–48.56%), and *Devosia* (2.14%–10.65%). Meanwhile, T1 was represented by 173 genera with the predominant genus being *Gemmobacter* (6.70%–26.38%), *Marivita* (5.53%–20.66%), *Rheinheimera* (1.75%–38.96%), *Hydrogenophaga* (4.8%–16.35%), and *Pseudomonas* (0.21%–25.81%). In contrast, treatment T2, with native *Bacillus* application, 202 genera were identified, with the most abundant genera being *Pseudomonas* (1.94%–57.27%), *Psychrobacter* (0.65%–38.99%), *Aeromonas* (0.07%–41.04%), *Rheinheimera* (0.13%–20.42%), and *Shewanella* (0.10%–34.75%). Throughout the study, these genera exhibited variations in their distribution and were not present on all sampling days, with the exception of *Devosia*, which remained present throughout the entire experiment. Collectively, the most abundant genera in CO and T1 were *Gemmobacter, Marivita*, and *Hydrogenophaga*. However, these genera were not represented in T2. Notably, T2 was the only treatment where *Bacillus* (0.59%−2.75%) was observed, with its abundance increasing progressively from day 02 and peaking at the end of the experiment (days 14 and 16). Interestingly, T2 also exhibited bacterial communities that were almost exclusive to this treatment and rarely observed in CO and T1, such as *Pseudomonas, Shewanella, Kluyvera*, and *Bacillus* (Fig 2B, S4 Fig, S5 Fig, S5 and S6 Table).

**Fig 3:**
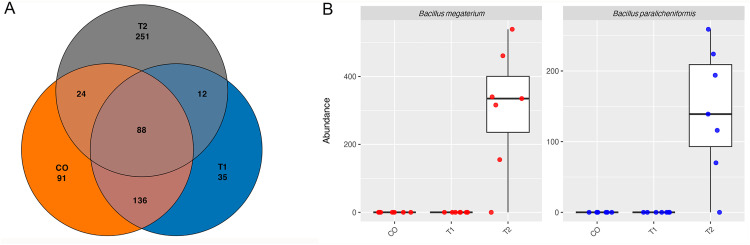
Comparative analysis of species in CO, T1, and T2 treatments. **A.** Venn diagram depicting the total ASVs shared at the species level between bacterial communities throughout the study for each treatment. **B.** Relative abundance of *Bacillus* species (> 100 reads) as exclusive species of the T2 treatment.

The overall distribution of shared and unique ASVs among the bacterial communities of the analyzed samples is shown in Fig 3A. A total of 88 species were identified as shared among all three analyzed groups. Treatment T2 exhibited the highest number of unique species, with 251 species in total, including *Bacillus megaterium* and *Bacillus paralicheniformis*, (Fig 3B) whereas CO and treatment T1 exhibited 91 and 35 unique species, respectively. When comparing pairs of groups, the CO and T1 shared the highest number of genera (136), while T1 and T2 only shared 12. This pattern was further supported by beta diversity analysis (S7 Table).

### 3.4. Alpha diversity of bacterial communities

Alpha diversity analysis was conducted to assess the richness and diversity of bacterial composition by evaluating well-known diversity indices such as Chao1, Shannon, and Fisher. The results from the Chao1 index revealed that, in CO, the lowest diversity value was observed on day 4 (101), while the highest values were recorded on days 10 and 13 (208.6 and 209.875, respectively). In treatment T1, the lowest value was recorded on day 10 (87), indicating a low species diversity. However, this value increased significantly on days 13 and 16, reaching 146 and 144, respectively, suggesting a much more diverse community towards the end of the study. In treatment T2, higher diversity values were observed at the beginning of the study on day 4 (216), with a decrease in diversity on days 10 and 13 (52). Overall, the Chao1 index indicated that the treatment T2 had the highest diversity, followed by the control, while treatment T1 was the least diverse.

The Shannon-Wiener index evaluates both species richness and the evenness of a microbial community, with values approaching zero in less diverse communities and increasing with higher diversity [42]. In CO, the index showed its lowest value (3.10) on day 2, indicating lower microbial richness, but increased to 3.81 on day 13, reflecting higher diversity and a more even distribution of species. In treatment T1, diversity remained relatively stable until day 14, when it dropped to 3.19, and then peaked at 4.10 on day 16, indicating a more diverse community. Treatment T2 exhibited the highest values in the early days, peaking at 4.49 on day 4, but dropped drastically to 2.14 on day 10. Overall, the Shannon analysis revealed that T2 showed the highest microbial richness, followed by T1 and CO, although by the end of the trial, T2 displayed lower evenness.

Fisher analysis estimated the species richness and distribution across the three conditions. In CO, day 13 showed a high value (36.88), indicating great diversity, while the lowest value was recorded on day 4 (15.45). Treatment T1 reached its peak on day 13 (23.94) and its minimum on day 10 (12.96), maintaining relatively constant values over time. In contrast, treatment T2 exhibited its highest richness and diversity on days 4 and 7 (38.39 and 34.97), with lower values on days 10 and 13 (7.11). Thus, T1 displayed more constant but lower diversity compared to CO, which stood out on days 10 and 13, while T2 showed more variable diversity throughout the bioassay (S8 Table).

**Fig 4:**
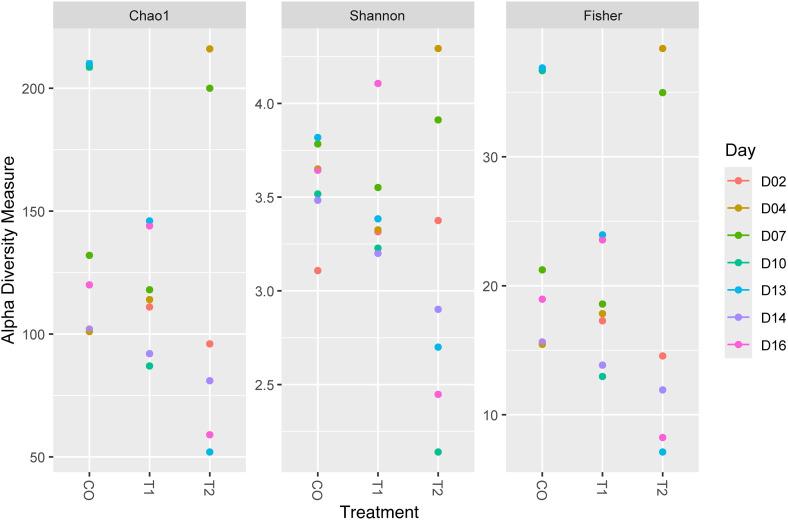
Alpha diversity profile of the bacterial community in low salinity culture water of *Litopenaeus vannamei.* The results, presented at the genus level, correspond to the communities from the control (CO), T1, and T2 treatments (p-value: 0.002; ANOVA F-value: 8.38).

The results of the Analysis of Variance (ANOVA) revealed a p-value of 0.002 and an F-value of 8.38, indicating significant differences in bacterial diversity between the treatments. The alpha diversity analysis showed that treatment T2 exhibited the highest microbial richness in the early stages of the experiment but experienced a notable decrease toward the end. In contrast, CO showed a continuous increase in diversity, reaching its peak on day 13. Treatment T1, although less diverse, maintained a relatively constant diversity throughout the study (Fig 4).

### 3.5. Beta diversity patterns of bacterial communities

**Fig 5:**
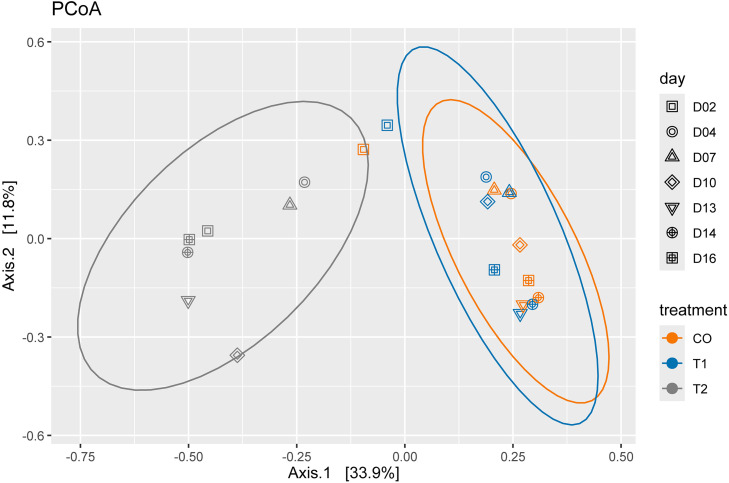
Principal Coordinate Analysis (PCoA) plot of Bray-Curtis distance of bacterial communities at the genus level in CO, T1 and T2 conditions, where shape indicates different days and color treatment (PERMANOVA between treatment, *P *= 0,001***, *R*^*2*^* = *0,328 with 999 permutations).

Beta diversity analysis was conducted to assess the effects of increased water exchange rate and the use of native bioremediation bacteria on the bacterial composition in low-salinity shrimp aquaculture water. Principal Coordinate Analysis (PCoA) showed that, among the 16 days analyzed, day 02 samples from CO and T1 were more closely related to T2 compared to the other days. Additionally, it was observed that the early days (04 and 07) clustered at the upper section of axis 2, while the later days (13, 14, and 16) clustered at the lower section. This pattern was more pronounced in CO and T1. However, day 10 did not exhibit a distinct grouping pattern with either the early or late days, with this difference being more noticeable in treatment T2 (Fig 5).

PERMANOVA analysis revealed significant differences (p = 0.001) among the three different conditions evaluated. To identify the most distinct conditions, we performed a pairwise PERMANOVA analysis, also based on Bray-Curtis distance. When comparing CO with T1, no significant differences were found (R² = 0.066, p = 0.561). However, the comparison with treatment T2 revealed statistically significant differences (R² = 0.32, p = 0.002*), S9 Table.

**Fig 6:**
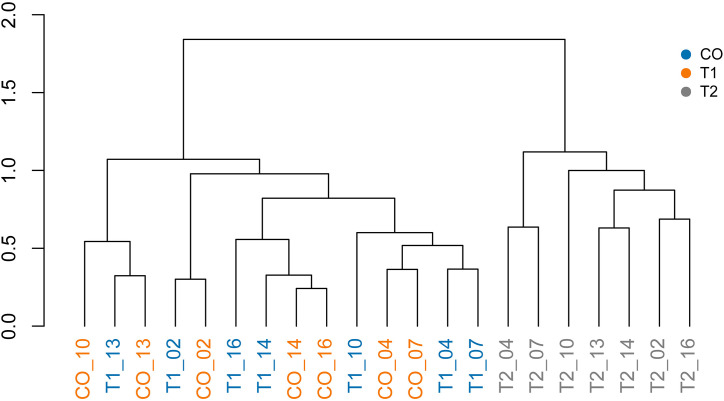
Ward’s hierarchical cluster analysis based on the Bray-Curtis dissimilarity matrix using relative abundance data from each sample in CO, T1, and T2 conditions. The colors represent each treatment, and the numbers indicate the day of sample collection during the bioassay.

In addition to the PCoA analysis, hierarchical clustering using the Ward´s method separated the three treatments into two main groups (Fig 6). The CO and T1 formed a cluster, while the second cluster consisted exclusively of T2 samples.

When analyzing the days, it was observed that in the T2 treatment, the early days (04 and 07) clustered into a single subgroup, while within the second subgroup, day 10 was distinctly separated from the others. Interestingly, the later days (13, 14, and 16), along with day 02, were grouped together. However, the first group, corresponding to CO and T1, did not exhibit clear internal subdivision, consistent with the pattern observed in the PCoA analysis.

The results of the Principal Coordinates Analysis (PCoA) and the PERMANOVA test revealed significant differences in bacterial composition among the treatments, with treatment T2 showing a distinct separation from both CO and T1. Additionally, hierarchical clustering analysis highlighted temporal patterns in bacterial composition, grouping the early days, except for the day 1, into a cluster that was distinct from later staged of the experiment.

### 3.6. Shotgun metagenomic analysis of genes involved in nitrogen metabolism

Considering that the native bacteria applied in T2 significantly reduced nitrate and nitrite levels in a sustained manner from day 10 onward, we compared T2 with CO in the shotgun analysis, as both groups were subjected to the same water exchange rate (25%).

**Fig 7:**
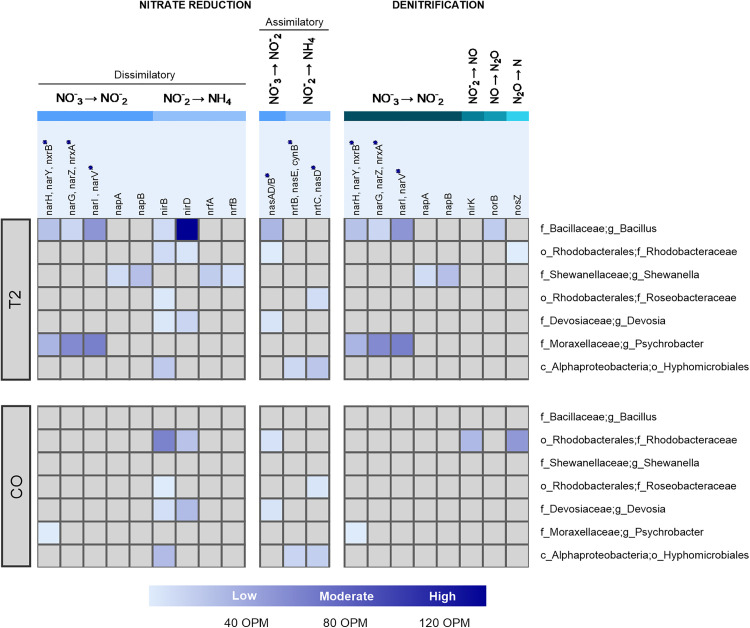
Heatmap showing the distribution of genes involved in nitrogen metabolism on day 10 for CO and T2. The abundances of the genes are presented in OPM (ORF per million). The OPM values of the genes were calculated collectively based on their orthology from KEGG.

In this context, the shotgun analysis results from day 10 in both CO and T2 were used to identify genes involved in nitrate transport, assimilatory nitrate reduction (ANR), dissimilatory nitrate reduction to ammonium (DNRA), nitrification, and denitrification (Fig 7, S10 Table).

The nitrate assimilation process begins with its transport into the cell via the NRT transporter. In this study, *NrtB* and *NrtC* genes were detected in both CO and T2, originating from Hyphomicrobiales and Roseobacteriales. Genes involved in assimilatory nitrate reduction, such as *NasAD/B, NasD*, and *NasE*, were identified in both treatment CO and T2, with the highest OPM values corresponding to the genus *Bacillus*. Notably *NasD* and *NasE* genes were present in comparable abundances in both conditions and were affiliated with Roseobacteriales and Hyphomicrobiales.

For dissimilatory nitrate reduction to ammonium, *narH, narY, narG, narZ, narI,* and *narV* genes from *Bacillus* and *Psychrobacter* were detected in T2, whereas in the control, only *narH* and *narY* from *Psychrobacter* were identified, with lower OPM values than those observed in T2. *nirB* and *nirD* genes, associated with intracellular ammonium production, were detected in both conditions with low OPM values in families such as Rhodobacteraceae, Roseobacteraceae, and Devosiaceae. However, in T2, these genes were exclusively found in *Bacillus*, with high OPM values.

The conversion of nitrate to nitrite was catalyzed by *NapAB* enzymes, while the subsequent reduction of nitrite to ammonium was mediated by *NrfA* and *NrfB*. These genes were exclusively identified in *Shewanella sp*. under the T2 treatment.

During denitrification, the genes *nirK, norB*, and *nosZ* were detected overall, although their occurrence differed between treatments In CO, *nirK* and *nosZ* from Rhodobacteraceae exhibited moderate OPM values, whereas in T2, *norB* from *Bacillus* and *nosZ* from Rhodobacteraceae were also present, with comparable OPM values.

## 4. Discussion

### 4.1. Application of *Bacillus* effectively reduces nitrite and nitrate concentrations in low-salinity shrimp farming water

The accumulation of nitrogenous compounds is a major challenge in intensive *L. vannamei* culture, as it can negatively impact pond production [43]. This problem becomes particularly critical under low – salinity conditions, where ammonium, nitrate, and especially nitrite, become more toxic as salinity decreases [8]. To address this problem, several biological strategies have been proposed, including the use of microalgae, biofloc, aquaponics systems, polycultures, consortia of autotrophic and heterotrophic bacteria, probiotics, biofilters, among others [44]. In this context, we evaluated the effect of two treatments on nitrogen compound removal in shrimp culture water at 4 ppt salinity: increased water exchange rate (T1) and the application of *Bacillus megaterium* and *Bacillus paralicheniformis* (T2).

The results demonstrated that in treatment T2, nitrite and nitrate concentrations stabilized at 0 mg/L from day 4 until the end of the trial (day 15). Similarly, the use of the L7 strain (*Bacillus methylotrophicus*) maintained nitrite concentrations at a stable level (<0.1 mg/L) starting from day 4 of the trial [45]. Other studies have also reported similar findings. For instance, the *Bacillus velezensis* AP193 strain maintained nitrite and nitrate levels below 0.1 mg/L [46], while the *Bacillus subtilis* A1 strain demonstrated high efficiency in nitrite removal by day 5 of the trial [47]. In contrast, CO and T1, which involved 25% and 50% water exchange, respectively, reached maximum levels of up to 5 mg/L for nitrites and 160 mg/L for nitrates. These values far exceed the recommended safety limits for *L. vannamei* (0.25 mg/L and 21.5 mg/L for nitrites and nitrates, respectively) [8]. Although no mortality was recorded in any of the three groups during the trial, such concentrations may compromise animal welfare and reduce long-term productivity [48]. The sustained removal of nitrites and nitrates observed in T2 can be attributed to the capacity of *Bacillus* species to perform heterotrophic nitrification and aerobic denitrification simultaneously [49–51].

Regarding total ammonia nitrogen (TAN) concentrations, although no significant differences were observed among treatments, all values remained within the safety levels reported for *L. vannamei* postlarvae reared at low salinities (≤ 0.81 mg/L at 1–3 g/L salinity) [8]. Nevertheless, T2 presented the lowest average TAN concentration (0.19 mg/L), with higher concentrations observed in CO (0.32 mg/L) and T1 (0.47 mg/L). These values are particularly lower than those reported in other studies evaluating *B. subtilis* and *B. licheniformis* on water quality in *Penaeus vannamei* culture, obtaining TAN concentrations ranging from 0.43 to 0.70 mg/L [18]. Thus, although ammonium accumulation is a relevant risk in systems where nitrogen transformations such as ammonification are intensified, in our experiment TAN did not accumulate to levels considered harmful for *L. vannamei* under the evaluated conditions.

The observations in T2 suggest greater efficiency in ammonium transformation, facilitated by the heterotrophic microbiota, which play an essential role in the nitrogen cycle [52]. Although traditional strategies such as water exchange or recirculating aquaculture systems (RAS) are widely used to control nitrogen compounds in aquaculture systems, they present important limitations. Water exchange demands high water resource consumption, while RAS systems tend to accumulate nitrates over time, potentially leading to chronic toxicity [53] or require the implementation of denitrification units.

In this study, it was observed that increasing water exchange (treatment T1) was insufficient to reduce nitrate and nitrite concentrations to safe levels, thus highlighting the limited effectiveness of this method in low-salinity systems.

Management has become imperative in aquaculture to reduce environmental impacts associated with excessive freshwater consumption [54]. In this context, the combined use of *Bacillus megaterium* and *Bacillus paralicheniformis* (T2) alongside minimized water renewal, has emerged as an eco-friendly and efficient alternative for controlling nitrogenous compounds. This water – conserving approach fosters more sustainable resource use of water and contributes to the mitigation of environmental degradation [55].

### 4.2. Efficiency of *Bacillus* isolated from saline environments and its application in freshwater shrimp farming

The use of living organisms such as bacteria, to convert harmful substances into non-toxic compounds is a sustainable process known as bioremediation [56]. A deeper understanding of the bacterial species employed in aquaculture systems can contribute to reducing their environmental footprint [57]. In this study, we evaluated *Bacillus megaterium* and *Bacillus paralicheniformis*, native strains isolated from mangrove soils, that previously demonstrated efficacy in the removal of nitrogenous compounds in seawater shrimp farming at 30 ppt [15], under low – salinity conditions (4 ppt). Although low – salinity may influence the aquatic microbiota, the exclusive detection of *Bacillus* species in treatment T2 suggests that these native probiotic strains remained and continued to exert a notable effect on the culture system. This result aligns with previous reports that showed the effectiveness of *B. licheniformis* at 5 ppt [58] and the successful use of *B. subtilis* at 15.8 ppt [59].

Although the use of bacteria to mitigate nitrogen compounds has been previously explored [60], this study provides new evidence regarding the adaptability of heterotrophic denitrifying bacteria isolated from saline environments [15] and their adaptation and successful performance under freshwater conditions. This supports their potential as a viable ecological strategy that, on one hand, lessen diseases and issues linked to seawater use [3] and, on the other hand, improves crop health while maintaining a more balanced aquaculture system [61].

### 4.3. Effect of water exchange and the application of *Bacillus* on bacterial abundance

In the present study, the abundance of the microbiota present in CO, T1 and T2, was analyzed through full 16S rRNA gene sequencing, enabling the identification of different taxonomic levels including families, genus, and species. *Gemmobacter* and *Marivita*, belonging the Rhodobacteraceae family, were among of the most abundant genera in the control and T1 treatments. This family has been commonly reported in shrimp culture water at various growth phases [43]. *Gemmobacter* is a widely distributed genus found in various habitats such as freshwater, marshes, forest ponds, and estuaries, and is often associated with coastal planktonic algae [62,63]. *Marivita*, frequently detected in biofloc systems [64] and super-intensive *L. vannamei* cultures [65], could play an important role in nitrogen removal. However, it has also been associated with mortality events in larval shrimp ponds [66]. Interestingly, *Marivita* was exclusive to the control and T1 treatments, which did not include native *Bacillus* addition. *Comamonadaceae*, another abundant family observed, inhabits the environment and exhibits anaerobic denitrifying activity [67]. *Hydrogenophaga* emerged as the most abundant genus in T1 and has been previously observed in biofilters of marine recirculation systems, where it contributes to sulfur oxidation [68]. *Devosia* was the genus with the greatest distribution on all sampling days for the three treatments and has been described as a potential bioremediator, found in habitats such as contaminated soils and beach sediments [69]. Moreover, *Devosia* exhibits ammonia and nitrite-oxidizing activities, which are favorable for nitrogeno compounds reduction in shrimp aquaculture [70].

The introduction of probiotic bacteria from the *Bacillus* strains in *T2* not only altered the abundance of predominant genera but also promoted the increase of less common bacteria in the CO and T1 treatments. *Bacillus* has been widely reported in aquaculture as a probiotic, improving water quality through the degradation of organic compounds and the reduction of nitrogenous waste [71]. Species such as *B. paralicheniformis,* used in this study, are recognized for their ability to reduce nitrites and purify water [72,73]. Furthermore, *B. megaterium* could have beneficial properties for the growth of aquaculture species, in addition to reducing ammonia levels [74,75]. Contrary to expectations, *Bacillus* represented only 0.59% − 2.75% in T2, suggesting that the introduced probiotics were not dominant in the culture water. This observation is consistent with the results of Zeng et al. [76], who reported that the addition of *Bacillus* spp. does not necessarily guarantee high abundance in habitats associated with *L. vannamei* culture environments. Several factors may account for this low representation, including biofilm formation within culture tanks, probiotic intake by the animals [15,77] and dilution effects in the aquatic environment [76].

Regardless of relative abundance, treatment T2 resulted in distinct microbial community structure compared to treatments that only received water exchange. Microbial community dynamics are influenced by a range of biotic interaction (species-environment) and abiotic factors (pH, temperature, dissolved oxygen, salinity, oxygen demand, and nitrogen levels) [78]. Nutrient supplementation can significantly alter the structure of microbial communities, as evidenced by Huang et al. [79], who demonstrated that glucose supplementation in the *L. vannamei* diet improved growth and survival, while inducing divergent responses in the microbiota with more pronounced variability observed in the water compared to the intestine. Other studies examining glucose supplementation suggest that the carbon-to-nitrogen (C/N) ratio could influence the diversity and structure of heterotrophic microorganisms in aquaculture systems [80,81].

Regarding bacterial abundance, the genus *Pseudomonas* was the most prominent in T2. The species *P. stutzeri*, *P. guineae*, *P. peli*, *P*. *putida*, and *P. yamanorum* (above 0.01) were identified, with *P. fluorescens* and *P. fragi* being the most abundant. These species are known for their capacity to perform heterotrophic nitrification and aerobic denitrification in freshwater and low – salinity environments [82–84]. Although most of the species mentioned are not classified as primary pathogens for shrimp some, such as *P. fluorescens*, may cause secondary infections under stress – inducing conditions [85]. Conversely the *P. fluorescens* strain NB14 has been evaluated for its beneficial roles in biofloc-based intensive aquaculture systems, including ammonia and nitrite removal, antagonistic activity against pathogenic *Vibrio* species, and immune enhancement [86]. Similarly, *P. stutzeri* has been considered a model for denitrification, innate immunity enhancement, and intestinal microbiota modulation [87,88]. Finally, *P. putida* has also been reported to remove ammonia in shrimp culture systems [84,89].

We report additional genera, included *Psychrobacter*, which are commonly distributed in marine and polar environments [90] and have shown probiotic potential in *L. vannamei* postlarvae [91]. Particularly noteworthy is the genus *Shewanella*, represented by *S. baltica*, which is considered an important bacterium in the reduction of nitrogen compounds [83,92]. This bacterium utilizes various electron acceptors, including nitrates and nitrites for energy acquisition [93].

The genus *Aeromonas* has been frequently reported as a pathogen in *L. vannamei* cultures [94,95]. At the species level, we identified *A. molluscorum* [96], *A. encheleia* [97], *A. rivipollensis* [98], *A. media* (Singh, 2000) [99] y *A. bivalvium* [100], which primarily affect mollusks and fish, with no well-documented impact on shrimp. We also identified *A. salmonicida*, which are predominant associated with mortality in fish [101], although it has been reported in crustaceans such as *Macrobrachium nipponense* [102] and *Metapenaeus affinis* [103]. Interestingly, we observed an abundance of 0.45% on day 10, which decreased to 0.24% on day 13 and was undetected on subsequent days. This reduction may be attributed to the ability of *Bacillus* species to alter the quorum sensing of Gram-negative pathogenic bacteria, providing an advantage for the control of aquatic infections [77].

In this study, it was observed that the total number of shared and unique bacterial genera varied significantly depending on the treatment applied. In this context, the control and treatment T1 shared a high number of genera, whereas T2 presented a large number of unique ASVs. These findings are consistent with those of Waiho et al. [104], who reported that, in a system with a 10% water exchange rate, the probiotic treatment shared 137 genera with the biofloc treatment, compared to only 17 genera shared with the control. However, it is important to consider that other variables, such as the mode of probiotic administration, may also exert an influence on microbial community composition [58]. Although the relative abundance of *Bacillus* in T2 was not predominant, both *B. paralicheniformis* and *B. megaterium* were found to be exclusive to this treatment.

### 4.4. Effect of water exchange and *Bacillus* application on bacteria diversity

It has been reported that water exchange exerts significant impact on the physico-chemical and biological factors of aquaculture systems, promoting bacterial proliferation through the accumulation of organic matter and nitrogenous compounds [105]. In the present study, it was observed that the control group exhibited a progressive increase in bacterial diversity, reaching its high levels on days 10 and 13. In contrast, treatment T1 maintained a relatively stable and lower bacterial diversity, which did not exceed the control. This suggests that as the water exchange rate increases, bacterial diversity tends to decrease slightly. These results are consisted with previous observation in systems with low water exchange (3%), where bacterial diversity was significantly greater compared to systems with high exchange rates (300%), which were predominantly colonized by ammonium- and nitrite-oxidizing bacteria [106]. Treatment T2, in contrast, displayed the highest bacterial diversity at the beginning of the study, followed by a gradual decline over time. Moreover, the microbial composition of T2 fluctuated more markedly in comparison to the control and T1 treatments. This outcome contrasts with reports from other studies, indicanting no significant differences in bacterial diversity between probiotic treatments and traditional biofloc systems without probiotics [107]. The results obtained, across all evaluated alpha diversity indices, suggest that the introduction of *Bacillus* sp. modulated the bacterial composition, resulting in a progressive reduction over the experimental period. The incorporation of *Bacillus sp.* in aquaculture systems has been shown to effectively prevent the growth of pathogenic bacteria [108], inhibiting them through the production of antimicrobial metabolites [109], which contributes to greater fluctuation in bacterial diversity.

Regarding beta diversity, our findings suggest that increasing the water exchange rate from 25% to 50% did not elicit substantial alterations in the bacterial community structure. Samples derived from the control and T1 groups showed high degree of similarity, particularly during the initial days, as reflected in the clustering patterns of the samples over time. These patterns align with prior studies reporting that microbial communities in systems with low and high water recirculation rates were more comparable to each other than those in systems with moderate recirculation [110]. Additionally, Deng *et al*. [111] attributed microbial variability to external environmental drivers such as salinity and dissolved oxygen, rather than hydraulic retention time, suggesting that these factors may have a greater impact on bacterial diversity, and consequently on crop health. Interestingly, our results also revealed notable distinctions between the CO and T2 groups, despite both employing a 25% water exchange rate. The addition of native bacteria with bioremediating capabilities resulted in a considerable shift in microbial community structure. A similar effect was observed in previous studies, which demonstrated that the use of probiotic bacteria, including *Bacillus* and *Gammaproteobacteria*, can significantly alter the microbiota of both water and the shrimp intestine [112,113], while *Bacillus licheniformis* notably modified bacterial populations in systems without water exchange [58]. These studies support the positive and significant role of probiotic bacteria on modulating microbial structure in aquaculture systems, potentially through bacterial competition between bacteria or the synthesis of secondary metabolites [114].

Regarding the persistence of the effects exerted by native bacteria, our results indicate that the application of *Bacillus* in low-salinity shrimp farming had a lasting effect on the water microbiota throughout the study period (16 days). Similarly, Vargas-Albores et al. [112] reported that the use of a commercial probiotic had sustained effects during shrimp cultivation (30–60 days). However, other studies suggest that this effect may diminish over time. For instance, Wu et al. [59] observed that the impact of *Bacillus subtilis* was more pronounced in the early stages of the cultivation period (day 16) but decreased towards the end (day 84). Likewise, Liu et al. [115] documented that the application of a commercial microbial agent significantly altered the microbiota in the early days (9–11), but these differences diminished towards the end of the study (days 14–21).

In our case, the impact of native *Bacillus* was sustained throughout the experimental period, suggesting that these bacteria confer a persistence benefit, in contrast to other treatments where the effects were observed to diminished over time.

### 4.5. Nitrogen metabolic pathways in Gram-positive and Gram-negative bacteria

Shotgun analysis identified genes involved in nitrate transport, assimilatory nitrate reduction (ANR), dissimilatory nitrate reduction to ammonium (DNRA), nitrification, and denitrification, revealing key microbial pathways in nitrogen cycling.

Nitrate uptake is initiated by an ATP-Binding Cassette (ABC) transporter, which typically comprises a cytoplasmic ATPase (*NrtD*), an ATPase/nitrate-binding fusion protein (*NrtC*), a periplasmic nitrate-binding lipoprotein (*NrtA*), and an integral membrane permease (*NrtB*) [116]. In our analysis, we detected *NrtB* and *NrtC*, suggesting that the microbial community has an effective mechanism for nitrate import.

Once inside the cell, nitrate can be metabolized through multiple pathways. Among these, the assimilatory nitrate reduction (ANR) pathway is particularly relevant, as it converts nitrate to nitrite via enzymes encoded by genes such as *NasAD/B, NasD*, and *NasE* [117,118]. Notably, *NasAD/B* showed higher operational protein mass (OPM) values in samples where *Bacillus* was predominant.

The DNRA pathway is mediated by nitrate reductases encoded by the narGHJI (*NarG, NarH, and NarI*) and narUZYWV (*NarY, NarZ,* and *NarV*) operons, both of which play key roles in nitrate reduction to nitrite [119,120]. In the present study, *narH, narY, narG, narZ, narI,* and *narV* were detected in T2, primarily associated with the genera *Bacillus* and *Psychrobacter*. In contrast, CO exhibited a more restricted genetic profile, with only *narH* and *narY* identified from *Psychrobacter*, and with lower OPM values compared to T2. These findings suggest greater genetic potential for DNRA in T2. Other key genes in the DNRA pathway encode the cytoplasmic nitrite reductase enzyme (NirBD), which is essential for intracellular ammonium production [121]. NirBD is active under aerobic conditions when nitrite is the only nitrogen source and has been suggested to regulate both the assimilatory and dissimilatory nitrate reduction to ammonium processes [60]. In our results, the *nirB* and *nirD* genes affiliated with the Rhodobacteraceae, Roseobacteraceae, Devosiaceae families and the Hypomicrobiales order exhibited low OPM values and were consistently detected in both the CO and T2 treatments. In contrast, these genes in members of the *Bacillus* genus were exclusively found in the T2 treatment and at high OPM values. This differential distribution suggests that *Bacillus* species may engage more actively in DNRA under the specific conditions applied in treatment T2, potentially reflecting an adaptive strategy to optimize nitrogen assimilation.

On the other hand, the conversion of nitrate to nitrite can also be catalyzed by the periplasmic nitrate reductase enzyme NapAB, a heterodimer in which NapA acts as the catalytic subunit and NapB as the electron transfer subunit [121]. Similarly, the NrfA subunit is responsible for reducing nitrite to ammonium [122], while NrfB facilitates the electron transfer from membrane-bound quinol dehydrogenase (NrfH or NrfCD) to the cytochrome c reductase of NrfA [119]. In this study, the NapA and NapB enzymes, as well as the *nrfA* and *nrfB* genes, were identified exclusively in treatment T2 and associated with *Shewanella sp*.

Meanwhile, during denitrification, the reductases encoded by *nirK* or *nirS, norB* *or* *NorC* and *nosZ* catalyze the sequential reduction of nitrate to nitrite, nitric oxide (NO), nitrous oxide (N_2_O) and finally to dinitrogen gas (N_2_) [123]. Although these key denitrification enzymes were detected across both CO and T2, their distribution differed between treatments. In CO, the *nirK* and *nosZ* genes from the Rhodobacteraceae family were observed with moderate OPM values, whereas in treatment T2, the *norB* genes from the *Bacillus* genus and *nosZ* from the Rhodobacteraceae family were detected with similar moderate OPM values.

Taken together, these results suggest that microbial communities in treatment T2 may be diversifying their nitrate reduction strategies. While the denitrification pathway was identified in both CO and T2, the exclusive detection of alternative enzymes in treatment T2 may indicate an adaptive response.

**Fig 8:**
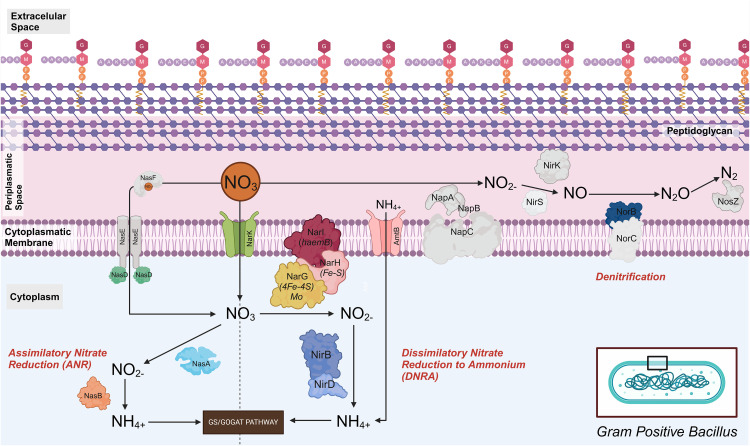
Proposed nitrogen metabolism pathways in Gram-positive bacteria *(Bacillus sp.)* based on metagenomic analysis. The dissimilatory nitrate reduction to ammonium (DNRA) and assimilatory nitrate reduction (ANR) pathways are illustrated. The genes identified in this study are shown in color, while the undetected genes appear in gray.

In this study, we propose metabolic pathway schemes identified in Gram-positive *(Bacillus sp.)* and Gram-negative bacteria in low salinity shrimp cultures, based on the genes identified through shotgun metagenomic analysis. From these genes, we developed a model for nitrogen compound processing in Gram-positive bacteria (*Bacillus sp.*). The nitrate entry into the cell is facilitated by the integral transport protein narK and the NasD protein of a nitrate transport complex. However, its associated proteins NasE and NasF were not detected. Once inside the cytoplasm, two pathways can be followed: (i) the assimilatory nitrate reduction (ANR) pathway, where the cytoplasmic protein NasA converts nitrate (NO₃⁻) to nitrite (NO₂⁻), followed by the action of NasB, which reduces nitrite to ammonium (NH₄⁺), and this is ultimately connected to the GS/GOGAT pathway, facilitating the reutilization of intracellular ammonium; and (ii) the dissimilatory nitrate reduction to ammonium (DNRA) pathway, which utilizes the membrane-bound proteins NarI, NarH and NarG to reduce nitrate to nitrite, and the proteins NirD and NirB to reduce nitrite to ammonium. Additionally, the transmembrane transporter AmtB could play a crucial role in the incorporation of extracellular ammonium, as visualized in Fig 8. It should be noted that only *norB* was identified in the denitrification process.

**Fig 9:**
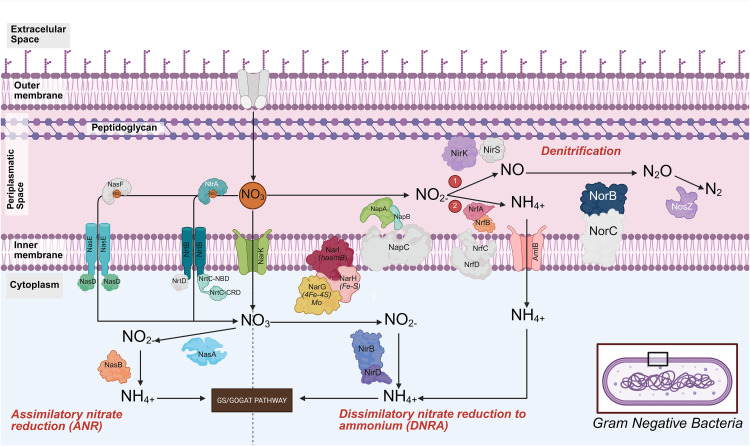
Proposed nitrogen metabolism pathways based on metagenomic analysis of Gram-negative bacteria. This figure illustrates the dissimilatory nitrate reduction to ammonium (DNRA), assimilatory nitrate reduction (ANR), and denitrification pathways. The genes identified in the study are shown in color, while the undetected genes appear in gray.

In Gram-negative bacteria, we identified the genes *NrtA, NrtB*, and *NrtC* from the Nrt transporter family, although the gene *NrtD* was absent. We also detected the transporters *NarK* and the Nas protein family (*NasF, NasE, and NasD*), which are responsible for nitrate transport into the cytoplasm. In the cytoplasm, nitrate can be metabolized through two principal pathways: (i) The ANR pathway, in which the protein NasA converts NO₃⁻ to NO₂ ⁻ , and NasB completes this pathway by reducing nitrite to NH₄ ⁺ ; and (ii) The DNRA pathway, in which cytoplasmic nitrate, once reduced by the protein NarG, part of the membrane complex narG/H/I, is converted to nitrite, and the proteins NirB and NirD catalyze the reduction to ammonium. Alternatively, we hypothesize that Nap may be involved in converting nitrate to nitrite, followed by the reduction of nitrite to ammonium through the periplasmic proteins NrfA/NrfB, coupled with their transmembrane proteins NrfC/D (non-identified in this study). Periplasmic ammonium could enter the cytoplasm via the AmtB transporter and would be utilized in the GS/GOGAT pathway (Fig 9). Additionally, we have identified genes related to canonical denitrification pathway, in which the proteins NapA/NapB convert nitrate to nitrite, followed by NirK or NirS, depending on the strain, which reduces nitrite to nitric oxide (NO), then the transmembrane protein complex NorC/NorB transforms NO into nitrous oxide (N₂O). Finally, NosZ completes the process by converting N₂O to molecular nitrogen (N₂).

In this context, we identified the assimilatory nitrate reduction (ANR) and dissimilatory nitrate reduction to ammonium (DNRA) pathways in Gram-positive bacteria. In contrast, Gram-negative bacteria exhibited these pathways along with the denitrification pathway. Nitrification and denitrification are key processes in nitrogen removal in aquatic systems. However, both processes have significant limitations. On one hand, nitrification, primarily carried out by autotrophic Gram-negative bacteria such as *Nitrosomonas spp., Nitrospira spp., Nitrosococcus spp*. and *Nitrosopumilus spp.* under aerobic conditions [124], can be affected by the slow growth of these bacteria and their sensitivity to environmental conditions. On the other hand, traditional denitrification, mainly performed by Gram-negative bacteria like *Paracoccus spp., Rhodothermus spp.,* and *Pseudomonas spp*., occurs only under anoxic conditions [125], limiting its application in certain treatment systems. In contrast to these traditional processes, heterotrophic microorganisms that perform simultaneous aerobic nitrification and denitrification (HNADM) can carry out both processes in the presence of an organic carbon source under aerobic conditions. This group includes genera such as *Acinetobacter, Bacillus, Cuprobacter, Halomonas, Klebsiella, Marinobacter, Pseudomonas, and Photobacterium* [12], mostly Gram-negative, except for *Bacillus*, which is Gram-positive and exhibits high genomic and metabolic adaptability.

Although the genes and enzymes responsible for nitrification in these heterotrophic organisms are less well characterized than in autotrophic bacteria and archaea (with key genes like *Amo* and *Hao*) [126], recent evidence suggests that some heterotrophic bacteria capable of ammonia oxidation encode their own ammonia monooxygenase (Amo), distinct from chemolithotrophic Amo. Furthermore, certain heterotrophic bacteria appear to oxidize ammonia independent of Amo activity, using membrane-associated oxidoreductases [127]. Our results show the presence of genes encoding nitrite oxidoreductase (*NxrA and NxrB*), involved in the second part of nitrification. However, the absence of identified homologs to *Amo* and *Hao* genes limits our ability to precisely determining the heterotrophic nitrification process. To overcome this limitation, future metatranscriptomic profiling could help us identify these genes.

In parallel, the GOGAT pathway is expected to mediate ammonium assimilation following its generation from other nitrogen sources [128]. Alternatively, ammonium may not be utilized within the cell and could be expelled. If utilized, two proposed mechanisms exist: facilitated transport via transmembrane proteins that act as transporters [129], or passive diffusion, utilizing the deprotonation of ammonium to convert it into ammonia, which facilitates this type of diffusion [130,131]. In our results, we found the presence of a single ammonium transporter, called AmtB. This transporter is present in both treatments, but with higher abundance in treatment T2. Several studies in *Bacillus subtilis* have highlighted its role in facilitated ammonium transport [132,133] and its connection with the GOGAT pathway by facilitating the entry of extracellular ammonium [128]. This could explain why ammonium levels (0.5 mg/L) remain low in treatment T2. However, the absence of significant differences in TAN levels among the three groups analyzed suggests that, although *Bacillus* does not play a primary role, its functional contribution may be crucial in explaining the assimilation of ammonium generated.

In our study, we observed that the enzymes identified in the metagenomic analysis of the CO and T2 on day 10 are primarily involved in the dissimilatory nitrate reduction to ammonium (DNRA) pathway and heterotrophic denitrification. In treatment T2, a higher abundance of enzymes associated with these processes was observed. Although this treatment evaluated the removal of nitrogen compounds using *Bacillus sp.,* the presence of genes encoding enzymes from other gram-negative bacteria, such as Shewanella and *Psychrobacter*, suggests an interaction between these bacteria in the transformation of nitrate and nitrite. These genera have been reported as DNRA bacteria and belong to the class Gammaproteobacteria, particularly in the orders Alteromonadales and Moraxellales, which are responsible for DNRA processes in various habitats [134].

The dissimilatory nitrate reduction to ammonium (DNRA) and heterotrophic denitrification are two key nitrate reduction pathways that interact and compete for both nitrate and dissolved organic carbon (DOC) [135,136]. The balance between these processes depends on multiple factors, including the C/N ratio (organic carbon), which is crucial for determining the competition between DNRA and heterotrophic denitrification. Numerous studies have reported that under anaerobic conditions, a high C/N ratio favors DNRA, producing ammonium as the predominant product, while a low C/N ratio favors denitrification [134]. However, recent studies conducted in *Pseudomonas putida* Y-9 have observed the opposite phenomenon in aerobic environments, where a low C/N ratio promotes DNRA, whereas a high C/N ratio favors denitrification [60]. In our study, the presence of the gene encoding the key enzyme of the DNRA process, NrfA, along with the C/N ratio of 0.175 (considered low) in treatment T2, suggests that the transformation of nitrates and nitrites would follow the pathway proposed for DNRA, without excessive ammonium release, with enzymatic contributions from both *Bacillus* and other gram-negative bacteria present in this treatment. For denitrification, the reduction of nitrate to nitrite follows a process similar to DNRA, but the nitrite is not reduced to ammonium, instead following a different pathway [137]. This pathway involves the enzymes nitrite reductase (NirS or NirK), which are considered genetic markers of denitrification [124], along with the enzymes Nor and NosZ, which reduce nitric oxide and nitrous oxide, respectively, and are involved in the formation of molecular nitrogen (N₂). Although denitrification typically occurs under anoxic or microaerophilic conditions, our study provides evidence that some bacteria are capable of converting various nitrogen species to N₂. In fact, recent studies have reported the existence of heterotrophic nitrification and aerobic denitrification (HN-AD) processes, which are responsible for denitrification in the presence of oxygen [138,139]. Hence, further investigation are necessary to identify key enzymes in these processes.

In this study, we observed the efficiency of *Bacillus* strains in reducing nitrites and nitrates during the 16-day evaluation period. However, the relatively short duration of the experiment constitutes a major limitation, restricting the assessment of long-term effects on shrimp production parameters. Additionally, the impact of *Bacillus* application on shrimp intestinal health and microbiota was not evaluated, representing another important limitation that could yield valuable insights into probiotic effects. Finally, the absence of transcriptomic analysis limits our understanding of the functional activity and gene expression dynamics of the microbial communities involved in nitrogen cycling. Therefore, future studies should incorporate the assessment of shrimp growth performance parameters, such as weight, size, and other indices related to water quality, over longer periods or throughout the entire shrimp farming cycle. It would also be interesting to explore the application of these *Bacillus* strains in other aquaculture systems, such as tilapia culture or RAS, and evaluate their effectiveness under different environmental conditions such as temperature, salinity, pH, and dissolved oxygen. Addressing these limitations will be essential to comprehensively understand and optimize the use of *Bacillus* strains in aquaculture.

## 5. Conclusions

This study demonstrates that the application of native *Bacillus* strains enhanced the bioremediation of nitrogen compound in low-salinity shrimp aquaculture. When combined with reduced water exchange, these strains maintained nitrite and nitrate concentrations at undetectable levels, outperforming conventional strategies based solely on water renewal. The application of *Bacillus* also reshaped the microbial community, favoring the dominance of genera such as *Pseudomonas*, *Psychrobacter*, and *Shewanella*. It also activated key genes involved in assimilatory and dissimilatory nitrate reduction. Although *Bacillus* species were not dominant in relative abundance, their exclusive detection in the treated system suggest a central role in these microbial and functional shifts. Furthermore, the absence of shrimp mortality during the study highlights the adaptability of these native *Bacillus* strains isolated from saline conditions (30 ppt) to low salinity shrimp farming (4 ppt). These findings support the use of native probiotic bacteria as an efficient and sustainable bioaugmentation approach for aquaculture management. Future research should explore their long-term impact on shrimp health and growth, microbiome stability, and applicability under varying farming conditions and system designs.

## Supporting information

S1 Table Nitrate, nitrite and TAN removal data. (XLSX)

S2 Table Quantification of DNA. (XLSX)

S3 Table Summary of Sequence Reads. (XLSX)

S4 Table 16S relative abundance at family level. (CSV)

S5 Table 16S relative abundance at genus level. (CSV)

S6 Table 16S relative abundance at species level. (CSV)

S7 Table 16S Venn diagram data at species level. (CSV)

S8 Table Alpha diversity at genus level. (CSV)

S9 Table PERMANOVA analysis. (XLSX)

S10 Table Annotation and assembly of the contigs. (XLSX)

S1 Fig Electrophoresis of genomic DNA. 1% agarose gel. CP 1,2 and 3: positive control. 1–21: samples. L1: Ladder 100 bp. L2: 1000 bp
(TIF)

S2 Fig Electrophoresis of PCR amplicons of 16S rRNA gene. 1.5% agarose gel. CP 1,2 and 3: positive control. 1–21: samples. L: Ladder 100 bp.
(TIF)

S3 Fig Relative abundances family level. (TIF)

S4 Fig Relative abundances genus level. (TIF)

S5 Fig Relative abundances genus level per treatment. (TIF)
