# Supplementary figures and images for: Bioaugmentation with native Bacillus strains enhance nitrate and nitrite removal and reshape microbiomes in low-salinity shrimp cultures: Elucidating genetic mechanisms
Source: PLoS One. 2026 Jan 9;21(1):e0339620. doi: 10.1371/journal.pone.0339620 (PMC12788651; doi:10.1371/journal.pone.0339620)

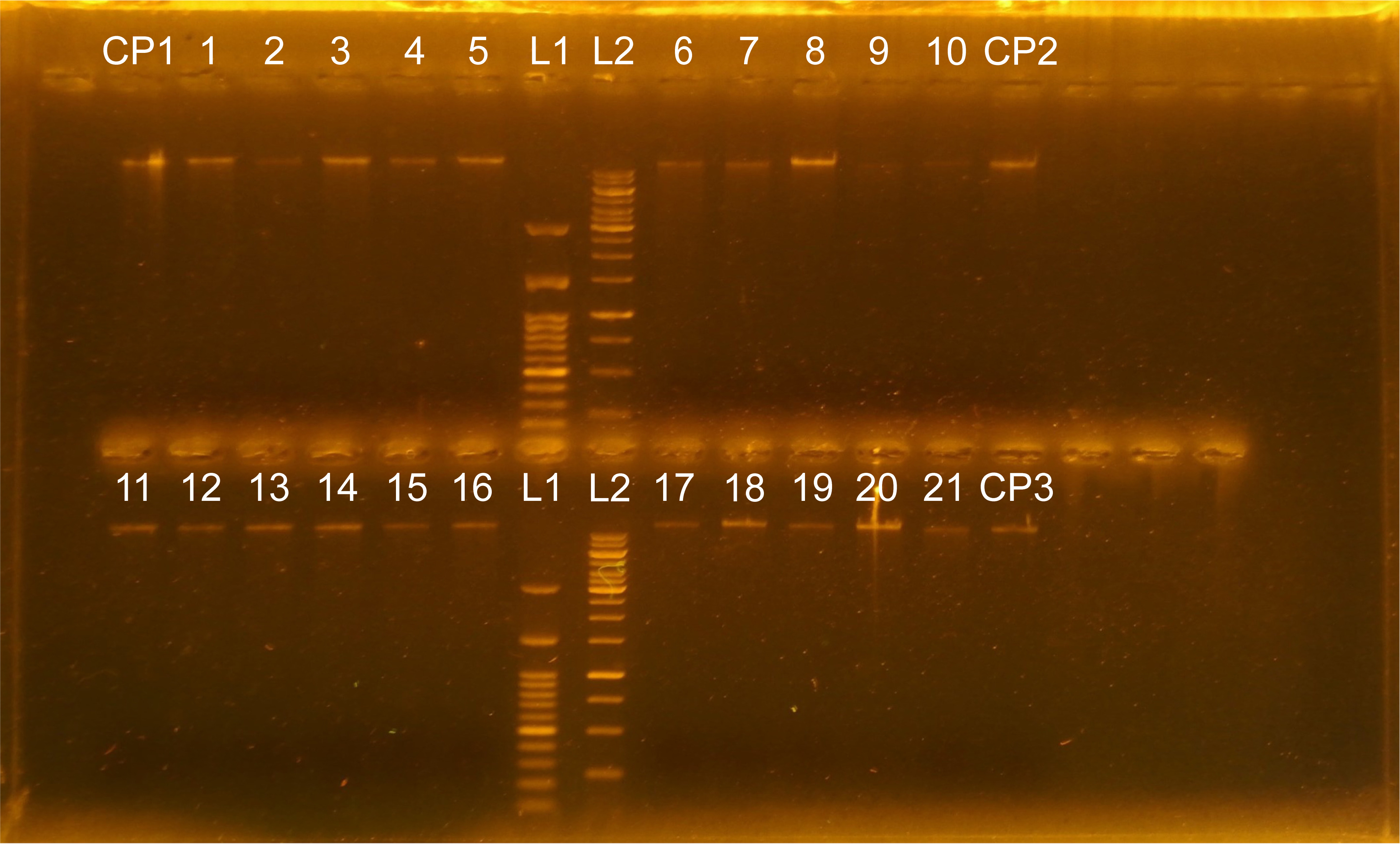

Supplement: S1 Fig — 1% agarose gel. CP 1,2 and 3: positive control. 1–21: samples. L1: Ladder 100 bp. L2: 1000 bp (TIF) [file pone.0339620.s011.tif]

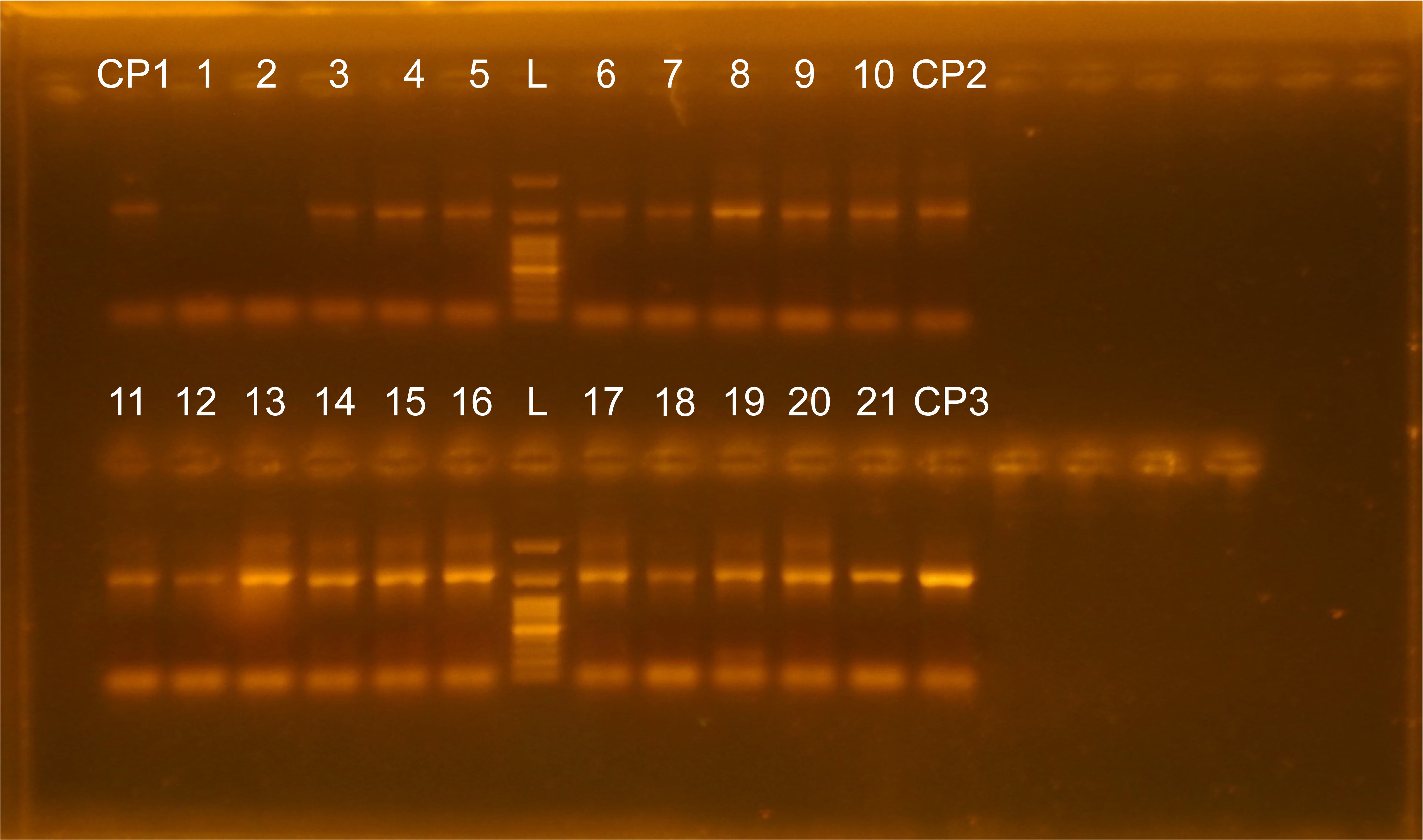

Supplement: S2 Fig — 1.5% agarose gel. CP 1,2 and 3: positive control. 1–21: samples. L: Ladder 100 bp. (TIF) [file pone.0339620.s012.tif]

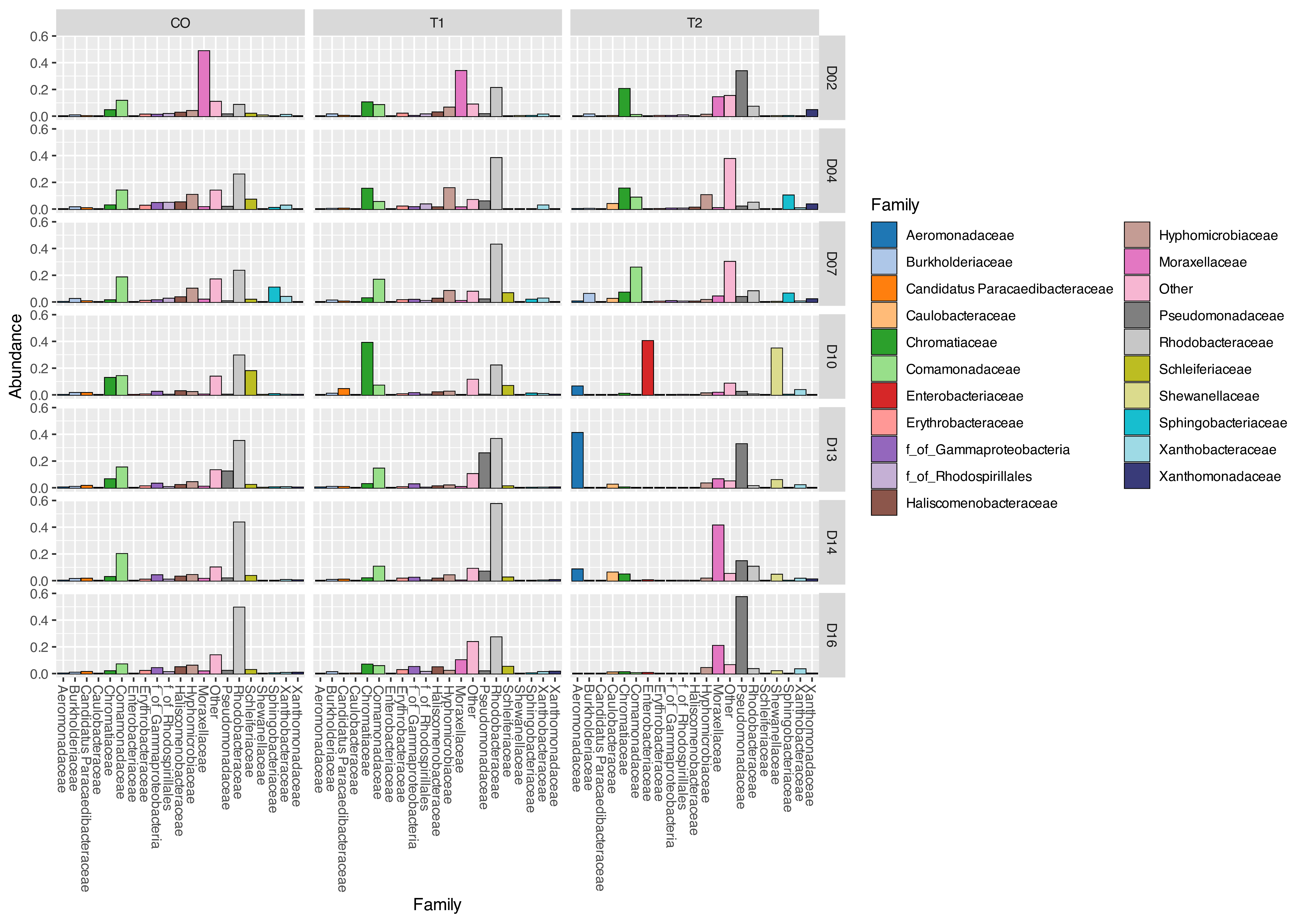

Supplement: S3 Fig — (TIF) [file pone.0339620.s013.tif]

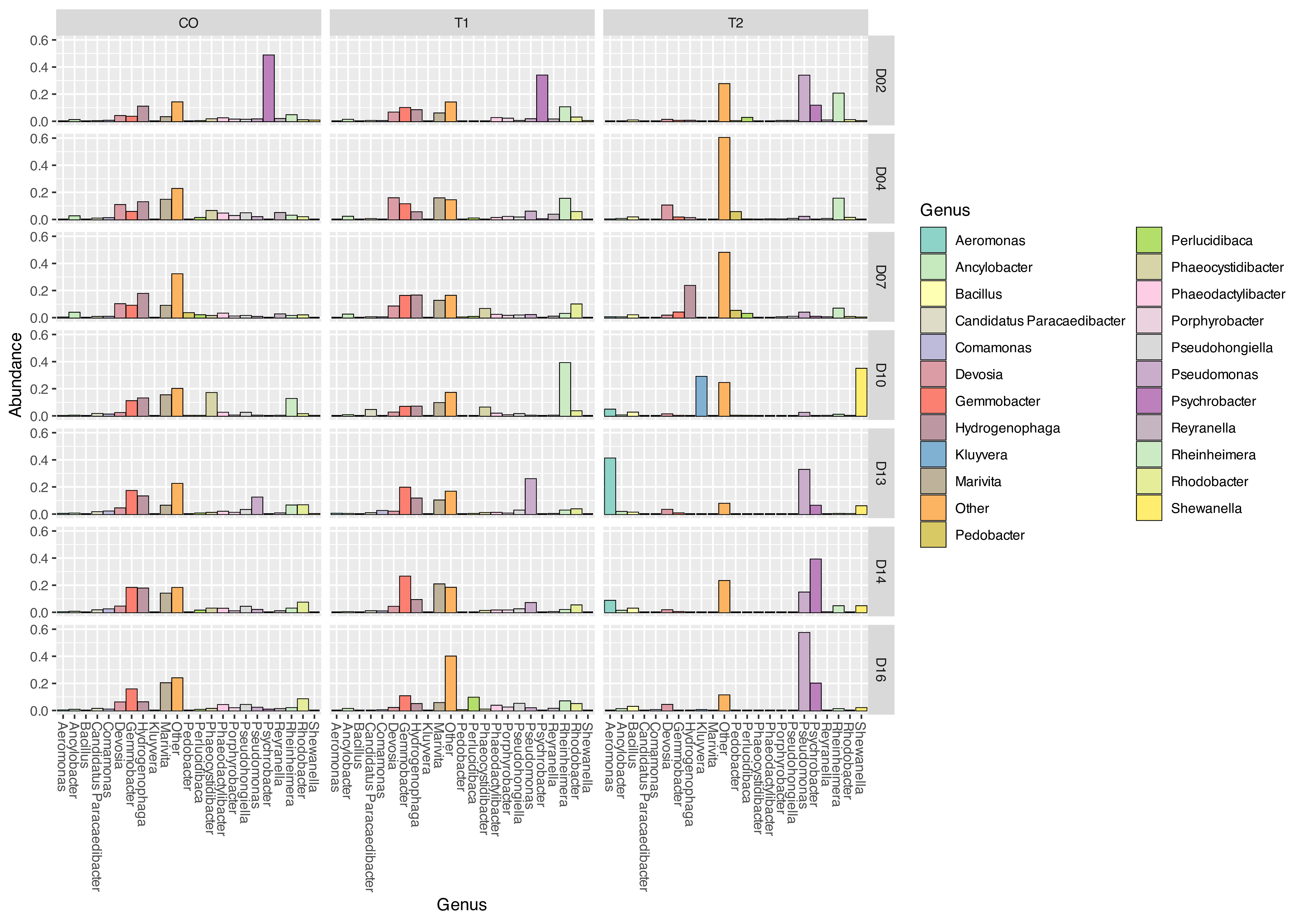

Supplement: S4 Fig — (TIF) [file pone.0339620.s014.tif]

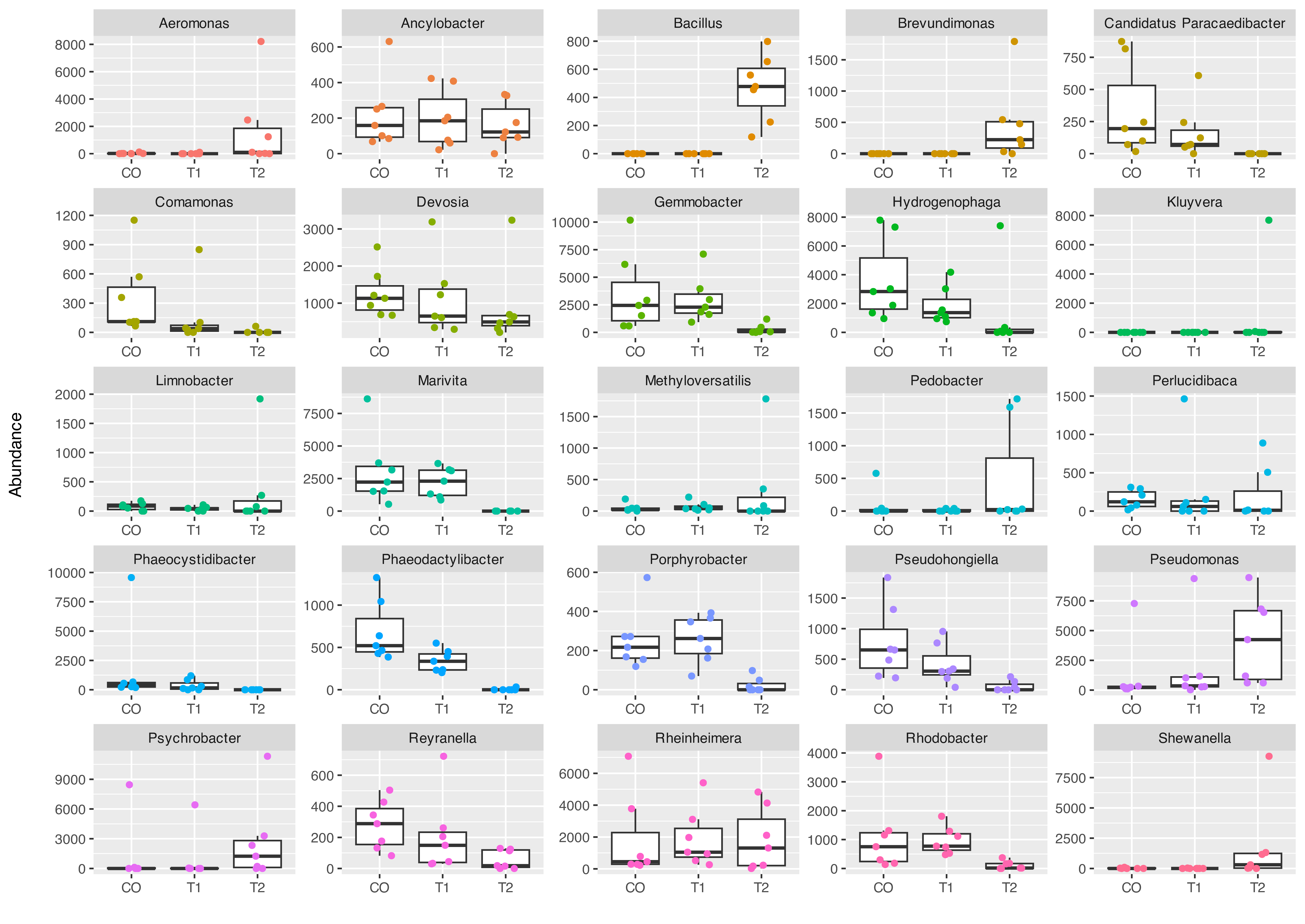

Supplement: S5 Fig — (TIF) [file pone.0339620.s015.tif]
